# Multilingual Framework for Risk Assessment and Symptom Tracking (MRAST)

**DOI:** 10.3390/s24041101

**Published:** 2024-02-08

**Authors:** Valentino Šafran, Simon Lin, Jama Nateqi, Alistair G. Martin, Urška Smrke, Umut Ariöz, Nejc Plohl, Matej Rojc, Dina Bēma, Marcela Chávez, Matej Horvat, Izidor Mlakar

**Affiliations:** 1 Faculty of Electrical Engineering and Computer Science, University of Maribor, 2000 Maribor, Slovenia; valentino.safran@um.si (V.Š.); urska.smrke@um.si (U.S.); umut.arioz@um.si (U.A.); matej.rojc@um.si (M.R.); 2 Science Department, Symptoma GmbH, 1030 Vienna, Austriatiefenbacher@symptoma.com (A.G.M.); 3 Department of Internal Medicine, Paracelsus Medical University, 5020 Salzburg, Austria; 4 Department of Psychology, Faculty of Arts, University of Maribor, 2000 Maribor, Slovenia; nejc.plohl1@um.si; 5 Institute of Clinical and Preventive Medicine, University of Latvia, LV-1586 Riga, Latvia; dina.bema@lu.lv; 6 Department of Information System Management, Centre Hospitalier Universitaire de Liège, 4000 Liège, Belgium; vchavez@chuliege.be; 7 Department of Oncology, University Medical Centre Maribor, 2000 Maribor, Slovenia; matej.horvat@ukc-mb.si

**Keywords:** multilingual framework, risk assessment, symptom tracking, chronic diseases, patient-centered care, real-world data

## Abstract

The importance and value of real-world data in healthcare cannot be overstated because it offers a valuable source of insights into patient experiences. Traditional patient-reported experience and outcomes measures (PREMs/PROMs) often fall short in addressing the complexities of these experiences due to subjectivity and their inability to precisely target the questions asked. In contrast, diary recordings offer a promising solution. They can provide a comprehensive picture of psychological well-being, encompassing both psychological and physiological symptoms. This study explores how using advanced digital technologies, i.e., automatic speech recognition and natural language processing, can efficiently capture patient insights in oncology settings. We introduce the MRAST framework, a simplified way to collect, structure, and understand patient data using questionnaires and diary recordings. The framework was validated in a prospective study with 81 colorectal and 85 breast cancer survivors, of whom 37 were male and 129 were female. Overall, the patients evaluated the solution as well made; they found it easy to use and integrate into their daily routine. The majority (75.3%) of the cancer survivors participating in the study were willing to engage in health monitoring activities using digital wearable devices daily for an extended period. Throughout the study, there was a noticeable increase in the number of participants who perceived the system as having excellent usability. Despite some negative feedback, 44.44% of patients still rated the app’s usability as above satisfactory (i.e., 7.9 on 1–10 scale) and the experience with diary recording as above satisfactory (i.e., 7.0 on 1–10 scale). Overall, these findings also underscore the significance of user testing and continuous improvement in enhancing the usability and user acceptance of solutions like the MRAST framework. Overall, the automated extraction of information from diaries represents a pivotal step toward a more patient-centered approach, where healthcare decisions are based on real-world experiences and tailored to individual needs. The potential usefulness of such data is enormous, as it enables better measurement of everyday experiences and opens new avenues for patient-centered care.

## 1. Introduction

With the increasingly aging population, episodic (symptom-triggered) healthcare is not meeting the needs of patients, especially those with chronic conditions [1]. In fact, in many cases it leads to poor health outcomes [2]. Namely, episodic care, even in those cases where early action has a direct impact on morbidity/survival, largely relies on the identification of relevant symptoms, and thus, on an individual to initiate the process. In fact, a radical shift toward person-centered care relies on assessing and responding to the self-reported needs of patients [3]. Patient-reported outcomes (PROMs) are increasingly used even in clinical practice [4,5]. Namely, PROMs can be used to identify problems and trace changes over time, especially in patients with (multiple) chronic conditions [6], by “monitoring” their health status or experiences using a set of standardized quantitative data collection instruments. PROMs consist of questions describing a wide variety of symptoms, side effects, functional changes, and quality of life [7,8]. Generic PROMs are designed to address a wide variety of patients and can be used to generalize/compare across multiple conditions [9]. Condition-specific PROMs have a greater validity, specificity, and responsiveness to changes in the patient’s specific condition [6].

It is obvious that the main challenge in the use of PROMs, from a systematic perspective, is to select the “right” PROMs and at the right time [9,10]. The selection must carefully reflect the intended purposes, including why the patient’s responses are collected (i.e., purpose), what is the goal of the investigation and what information it needs to collect, and how this information will be used to benefit the individual [11,12,13]. Due to the complexity and individuality of a “perfect” list of questions, patients do not all benefit equally from their use; some engage minimally, and some do not engage with PROMs at all [14]. In many cases, the PROMs do not address, or only partially address, the most apparent issues experienced by the patients or concepts that matter to underserved groups [15]. This creates a discrepancy between the perceived personal benefits and the effort required for the intervention [14]. The lack of proper translation or help with navigation leads to inaccurate captures of data and makes completion of the measures burdensome and challenging [16,17]. Furthermore, repeatedly answering the same questions about symptoms and general health can contribute to an increased focus on the disease, causing anxiety and emotional distress [18]. Finally, even when PROMs are fully completed and the completion rates are high, data validity depends on comprehension and patients’ ability to select responses that accurately reflect their experiences [19]. Furthermore, their validity is in general limited within a relatively homogenous diagnostic group. The tools become less reliable in more general populations and underrepresented subgroups (e.g., adoption groups, immigrant groups, disability groups) [20,21]. Overall, PROMs are a gold standard in the design of symptom diaries (i.e., structured, closed-ended questionnaires to collect symptoms) and provide a reliable tool for screening and identifying symptoms of diseases with a certain degree of confidence [22,23,24]. There is, however, a concern that symptom diaries may be subject to multiple biases, e.g., poor recall and timing bias, fatigue bias, collection mode-related bias, relevance bias, etc., and thus may be insensitive [25,26,27,28,29]. As a result, they are often the subject of careless reporting and may trigger a significant number of false positives and negatives [30,31,32]. Finally, symptom-based screening can induce additional stress and anxiety, especially in populations with high risk [33]. Personal digital diaries, on the other hand, may represent a more sensitive tool, especially for prescreening and assessing treatment responses [29,34]. Namely, because people provide frequent reports on the ”important” events and experiences of their daily lives, personal diaries offer a unique window on human phenomenology [35]. Furthermore, due to the subjective nature of symptoms, the semi-structured and open-ended designs of such diaries encourage individuals to identify what to record (report) [36,37]. The ability to use their own words and understanding may significantly decrease the perception of “wrong” questions and thus have a potential to significantly increase adherence and the quality of reporting.

Overall, digital diaries and digital screening tools (ePROMs) represent promising means of collecting real-time information and following people with complex (chronic) conditions [38,39,40]. However, to be truly valuable, weaknesses such as poor data utility and lack of in-depth information, patient burden and compliance, recall bias and diary fatigue, no continuous quantification, rigorous training in a challenging population, etc., need to be overcome [39,40,41,42,43,44]. The main motivation behind this paper is the efficient collection of high-quality, near real-time information, with significantly decreased complexity and burden of reporting. The framework simulates “doctor-patient” communication, i.e., creating an initial assessment and providing follow-up symptom-specific questions. We first deployed a pre-screening tool, which can collect an initial set of symptoms from speech. The response was analyzed and possible symptoms and causes extracted using a natural language processing tool. Using the symptoms and causes, we created a symptom-specific discourse to generate more in-depth information and insight. At the same time, the discourse ensures consistent quality of reporting. Finally, the information collected can then be used to trigger disease-specific (e)PROMs, when required, to further mitigate the self-reporting bias. Thus, the proposed tool also optimizes the use of PROMs and requests them from the patients on a necessity basis.

The paper is structured as follows. First, we represent the technology of the MRAST framework, which includes the mHealth patient and clinician app; the MSN, which includes the automatic speech recognition (ASR) SPREAD system [45]; and application of the Symptoma AI, emphasizing the utilization of Symptoma’s proprietary technology for extracting medical risk factors. The technology’s proficiency has been established in previous studies [46,47,48,49]. The MRAST framework also includes the FHIR server with implementation of the HAPI FHIR server [50] to store the patient data. Second, the paper provides insights into connectivity tests, elucidating the system’s robustness and scalability. Third, the patient evaluation section sheds light on the real-world usability of the integrated technologies, as reflected through general patient feedback and three rounds of a self-defined, patient-centered survivorship care plan after cancer treatments, based on big data and artificial intelligence technologies (PERSIST) block ABC surveys with questions related to the mHealth app, video diaries, and chatbot questionnaires. Section 4.3, Patient Evaluation, includes some of the results related to the MRAST framework. System usability scale (SUS) surveys were also provided to grade the mHealth app for patients and clinicians. Finally, the discussion section interprets and contextualizes the obtained results, exploring the implications of the MRAST framework. The paper concludes by summarizing the key findings and offering perspectives on the future implications and enhancements of the integrated technologies in healthcare.

This paper contributes multiple results, as presented in the results section. The MRAST framework was developed to provide a convenient and nonintrusive way of collecting, integrating, and representing patient-collected real-world data and outcomes in the form of structured questionnaires and video diaries. The paper presents the connectivity tests with the Symptoma AI, which provided the proposed symptoms and causes based on the ASR results, and we measured the resource’s consumption. Patients graded the use of the mHealth app, questionnaires, and video recordings, and this paper shows their responses.

## 2. Related Work

There are multiple technical implementations of solutions for collecting ePROMs being used in practice more and more often, ranging from web platforms [51,52,53,54,55] to dedicated mobile solutions [56,57,58,59]. Mobile solutions are observed as more practical, yield higher response rates, and result in fewer missing items [60,61]. With the digital PROMs offering many advantages over paper-based collection, they are preferred also from a practical perspective; e.g., they offer improved data quality, faster completion time, and decreased costs of data collection [62]. As already highlighted in the introduction, the main disadvantages of ePROMs from a patient’s perspective, and reasons for patients not using ePROMs, can be summarized as (i) ability to use (e.g., physical ability due to health issues), (ii) engagement (e.g., patients do not find them relevant because no symptoms exist), (iii) technical issues and usability (e.g., low technical proficiency), (iv) data security and trust [14,52]. Specifically tackling technical issues and usability, chatbots have been an efficient solution to improve usability and simplify the app functionalities and user experience [45,59,63,64,65]. Namely, chatbots exploit artificial intelligence and natural language processing to interact with patients without human intervention. At the same time, they can personalize the engagement, decrease the complexity compared with standard computer-based surveys, and overall offer a more “human-like” natural language collection of information [66]. The response rates are still relatively low [67]. The reasons for the low response rates are best summarized as disinterest, lack of time, inability to comprehend the questions, and anxiety [68].

Also from a practical perspective, the main barrier to a sustainable collection of PROs relates to engagement, which denotes disinterest and lack of time and comprehension. The digital diaries can improve the patient experience and decrease anxiety [69]. Using semi-structured and unstructured digital diaries with open-ended questions (i.e., interviews) represents an efficient alternative method of collecting patient experiences and outcomes [70]. The diaries with open questions represent collection of data that is unstructured in nature [71]. Overall, the nature of the information captured in such semi-structured interviews with open questions (i.e., in-depth interviews) offers the most reliable means of soliciting information from patients, from multiple perspectives [72,73]. However, when not conducted face-to-face, significant data loss could occur [74]. Namely, the method of in-depth interviewing is carried out as an interview guided by a flexible interview protocol and supplemented by follow-up questions, probes, and comments [75] that make the conversation focused and flexible and prevent poor or limited responses. Recently, there has been a growing interest in AI-enabled chatbot-based systems [76,77].

The chatbot technology could potentially mimic clinical interviews as specific activities such as health surveys, retrieving and analyzing health data, or the translation of diagnostic patterns considering behavioral indicators such as physical activity, sleep, nutrition, etc. Regarding data collection from patients, a plethora of research analyzed the use of chatbots in place of traditional form-based surveys and traditional (e)PROMs [78,79,80,81]. In fact, with closed-ended questions (which PROMs essentially are), the chatbots collect the same quality, if not higher, compared with digital surveys [33,82]. The solutions conducting interviews with open-ended questions generate more noise (less precise data); however, compared with the closed-ended solutions, their participants showed higher engagement and provided higher-quality responses when engaged with the chatbot [83,84,85]. With the recent breakthrough in large language models (LLMs), e.g., OpenAI’s GPT3, Google’s PALM, Gopher, Bing Chat, and Azure Health Bot, etc. [86,87,88,89,90,91,92], the technology has truly led to the development of powerful AI chatbots capable of engaging in natural and human-like conversations. In fact, LLMs are rapidly advancing to achieve near human-level performance on medical question-and-answering benchmarks [93,94]. As a main barrier to their facilitation in the health domain, the LLMs reflect the biases inherent to the systems they were trained on, i.e., the human interactions and internet data. This means their use can lead to manipulation, deception, and can even damage the users’ trust and negatively impact the users’ well-being [95,96]. Furthermore, the LLMs fail significantly when faced with a list of specific questions [97].

To sum up, it is well-acknowledged that incident reporting under-represents the actual frequency of events [98]. A significant part of this can be attributed to the effects of hindsight and outcome bias and the ability to discern “right” from “wrong” [99]. This is further emphasized by individuals’ ability to express themselves and articulate specific issues [19]. In this paper, we propose using a semi-structured, short questionnaire capable of capturing participants’ perspectives regarding an experience or an issue using their own words [100], eliminating the issue of comprehension. We describe a solution to collect patient self-reports using a storyline based (not LLM) speech-enabled chatbot, where the storyline is built dynamically based on symptoms expressed by the patient during each open-ended question. This means that the open-ended questions can be followed by a series of closed-ended questions, targeted to provide further context on the symptoms expressed by the patient. Compared with LLMs, the solution is limited in terms of discourse diversity; however, it does not introduce bias or responses potentially dangerous/negative to end users. Furthermore, it extends the traditional open-ended medical chatbot solutions with the capacity of extracting symptoms and causes from user responses and thus extending the interview with follow-up questions, and compared with traditional (e)PROMs, modeling the discourse to be relevant to actual issues experienced by the patient. Regarding the evaluation of user experience, we have found and compared some studies [101,102,103,104,105] that also include mHealth apps for cancer patients. The comparison of those studies is presented in Section 4.4, Feasibility of MRAST Framework in the Real World.

## 3. Methodology

The methodology section of this study presents a comprehensive approach known as the MRAST framework, designed to harness the power of decentralized architecture and advanced technologies in the context of healthcare. This framework comprises several key components, each serving a unique role in enhancing patient care and generating valuable insights. In this introduction, we delve into the fundamental aspects of the MRAST framework, with a focus on its primary elements, including the PERSIST mHealth application, a multimodal sensing network, and the big data platform based on Open Health Connect (OHC). Additionally, we explore the speech recognition engine, which plays a crucial role in extracting information from diary recordings. The methodology also highlights the transformation of diary recordings into updated patient profiles and the utilization of Symptoma’s AI to mine valuable information from the extracted data. Furthermore, it outlines the role of the big data platform and the HL7 FHIR server in managing and integrating patient information from various sources. Overall, the MRAST framework represents an innovative and holistic approach to healthcare, combining cutting-edge technologies and data-driven insights to improve patient well-being and disease management.

### 3.1. Environment

**Figure 1:**
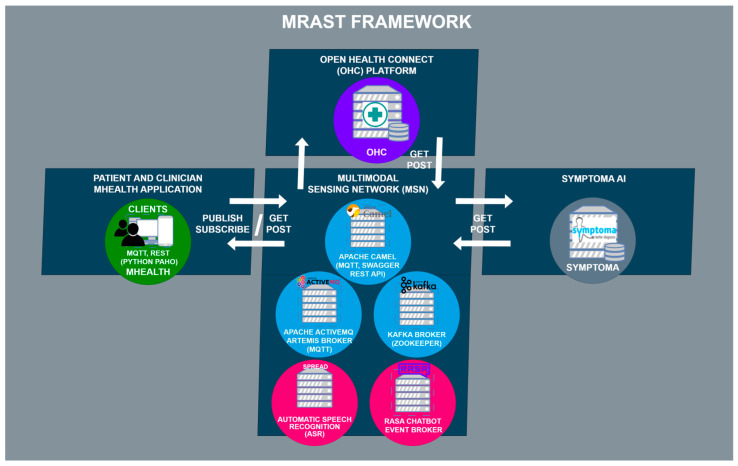
The architecture of the MRAST framework.

Figure 1 shows the MRAST framework as a whole. The MRAST framework consists of the mHealth patient and clinician apps, OHC platform, Symptoma AI, and MSN. The MSN’s architectural structure can be broken down into three components: Apache Camel, Apache ActiveMQ Artemis, and Apache Kafka. Apache Camel serves a dual role as both an external access point to the MSN and an internal link between Apache ActiveMQ Artemis and Apache Kafka. It operates as a Spring Boot application with an embedded Apache Tomcat server, facilitating HTTP requests. This Spring Boot application runs on a virtual machine and functions as a router, enabling the seamless exchange of data across various protocols.

Apache ActiveMQ Artemis operates as an MQTT broker, serving both internal and external connections to the mHealth app, which subscribes to specific topics. On the Apache Camel side, a REST API is implemented using REST DSL with Java, complemented by Swagger UI (OpenAPI) for documentation and REST endpoint testing. The mHealth app communicates with the Apache Camel REST API for managing questionnaires and user validation. The integration between Apache Camel and Apache ActiveMQ Artemis occurs through two methods: one via the Java Messaging Service (JMS), functioning similarly to MQTT with its support for topics and queues. Meanwhile, Apache Kafka, a distributed event streaming platform, plays a vital role in the MSN, managing data distribution through AI-based microservices.

MSN is tasked to enable communication between the components outside of the MSN, which include the patient and clinician mHealth apps, the Open Health Connect platform with FHIR server, and Symptoma’s AI.

### 3.2. The MRAST Framework

**Figure 2:**
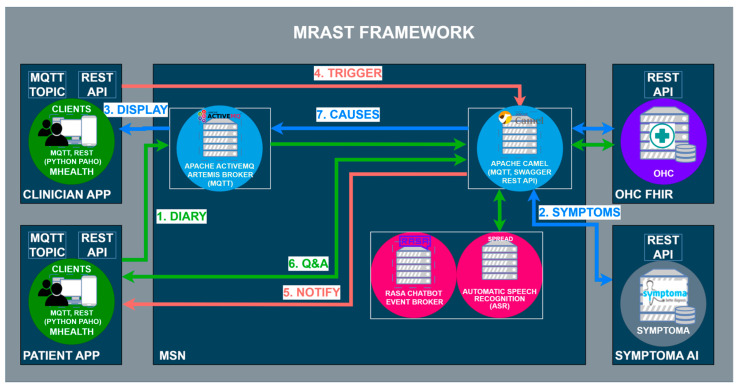
Overall architecture of the MRAST framework.

The MRAST framework in Figure 2 is built on a fully decentralized architecture that consists of four main components: (i) the PERSIST mHealth application, serving as the main interface for patients and clinicians; (ii) a multimodal sensing network, delivering software sensors to extract symptoms and causes; and (iii) the big data platform based on Open Health Connect (OHC) [106], a digital platform that provides the building blocks of connected health and care systems. The mHealth application consists of an application for patients and an application for clinicians. Within the scope of the MRAST framework (Figure 1), the role of the application for the patients is to enable and guide the diary recording process (1), deliver notifications (5), and display the disease-centric discourse (DCD) (6). Within the scope of the MRAST framework, the role of the application for clinicians is to display symptoms from the diary (2, 3), allow them to trigger the DCD (4), and finally, review the results and possible causes of the symptoms (3, 7). The process starts with the patient doing a video recording. In the mHealth application, the patient gets a notification to record a video diary. Once the patient taps on the notification patient, they will be led to a screen where the patient can start the recording. When the patient finishes with the recording, the video is stored to an OHC server and linked to the FHIR server resource as a link. Over the link, the MRAST framework can access it to process it further. From the video recording, the MRAST framework then extracts the symptoms. The second part is the collection of the questionnaire responses to obtain detailed specifications of symptoms. Over the mHealth application, each patient gets a notification over the MQTT protocol to fill in the questionnaire. Once the user taps on that notification, the questionnaire opens, and the user answers the questions until the end. For providing the new questions and retrieving the patient answers, we implement the REST protocol with a Rasa-based chatbot. Collected answers on questions are stored to the FHIR server when the user answers the last question.

The multimodal sensing network [107] represents the “brain” of the MRAST framework. It consists of components and end-to-end services to facilitate the symmetric interaction [45], including the speech recognition engine SPREAD, speech synthesis framework PLATOS, natural language services (including a Rasa-based chatbot), and conversational language generation services, i.e., the embodied virtual agent framework EVA [108]. Additionally, the framework integrates a symptoms extraction and tracking framework, which includes a depression classification pipeline and a risk assessment component built on top of Symptoma AI [47,109] for extracting clinical cues from free text, assessing risk factors, and returning risk scoring. MSN is a microservice-based component where the services are running as a virtual machine on an Ubuntu-based server that runs the Proxmox Virtual Environment. Some of the services, mostly the ones that need the use of GPUs, are running directly as services on Ubuntu-based servers with GPU cards (2x NVIDIA GeForce RTX 3050 Ti) used for inference. MSN is protected with the implementation of the VPN and firewall that allows specific ports to specific IP addresses with the use of SFPT and SSH to access the files and the commands terminal.

Finally, the OHC is a complete integration and streaming platform for large-scale distributed environments. Unlike a traditional messaging system or stream processing API, OHC enables all interfaces to be connected to and make decisions across disparate data sources in real time. OHC provides the framework and set of tools for the integration, ingestion, storage, indexing, and surfacing of patient information. The OHC platform is also a microservice-based platform where services are mostly running as Docker containers. The main component is the HL7 FHIR server, the Keycloak identity and access management service that generates the JWT token for safe access to the data that are stored on the FHIR server. OHC also offers a Kibana search that was used for stored data representation in the form of graphs. OHC is explained in more detail in Section 3.2.3, Big Data Platform and HL7 FHIR Server.

#### 3.2.1. Speech Recognition Engine

**Figure 3:**
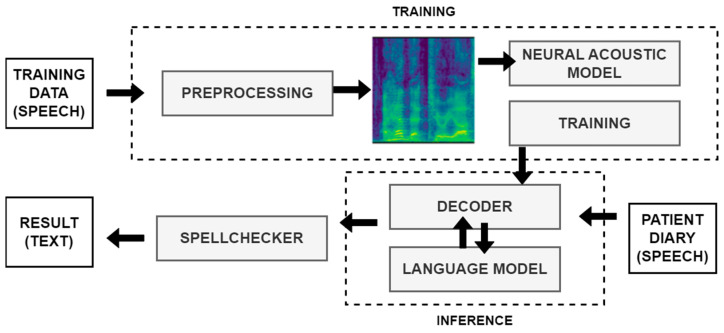
ASR SPREAD: an end-to-end architecture.

To extract speech from diary recordings, we deploy an end-to-end multilingual automatic speech recognition (ASR) system, SPREAD [45]. SPREAD is built on an end-to-end connectionist temporal classification-based deep neural model. The acoustic model is based on the B × R Jasper model [110]. In SPREAD, we extend it with a natural language model and spell-checker. To facilitate the challenges of the “data in the wild” [111], the system includes a spell-checker model and a 6-gram KenLM [112]-based language model. The overall architecture is outlined in Figure 3.

In this end-to-end ASR model, acoustic and pronunciation models are replaced with a convolutional neural network (CNN). In the preprocessing phase, the mel filter bank features are calculated from 20 ms windows, and a 10 ms overlap is used. The ASR engine outputs a probability distribution over characters per frame. The engine has a block architecture; therefore, the B × R model has 10 blocks, each with 5 sub-blocks. Several operations are applied to each sub-block, such as a 1D-convolution, batch norm, ReLU, and dropout. Within each block, all sub-blocks have the same number of output channels. There is a residual connection between each block, which consists of a projection layer followed by batch normalization. The NovoGrad [113], an optimizer similar to Adam [114], is used to compute second moments per layer instead of per weight.

The decoder converts a probability distribution over characters into text. There are different types of decoders that are usually employed with CTC-based models: greedy decoder and beam search decoder, with or without spell-checker model, with or without language model re-scoring, etc. A greedy decoder outputs the most probable character at each time step. It is very fast, and it can produce transcripts that are very close to the original pronunciation. However, it may introduce many small misspelling errors. Due to the nature of the word-error-rate (WER) metric, even one character error makes a whole word incorrect. Thus, a beam search decoder with language model re-scoring and spell-checking allows for many possible decodings (beams) at once, assigning a higher score for more probable N-grams according to a given language model. The language model helps to correct misspelling errors. The downside is that it is slower than a greedy decoder.

The spelling correction (SC) models are used to explicitly correct acoustic ASR errors. In SPREAD, we are utilizing text-only data by training a supervised “spelling correction” model to explicitly correct the errors made by the acoustic model. Instead of predicting the likelihood of emitting a word based on the surrounding context, as in RNN-LM [115], the SC model in SPREAD only identifies likely errors in the acoustic model and proposes alternatives. We integrate a context-aware spell-checking library for automatic spelling correction. Correction and error detection targets to correct up to three edit distance errors and splits two merged words when needed. The SC uses a combination of CatBoost gradient-boosted decision trees, N-gram language models, and a static dictionary for error detection and candidates ranking. For each word, a set of features is generated, such as word length, prediction of 2-gram lm, 3-gram lm, 4 masked gram, absence or presence in the dictionary, and others. A fast classifier makes a prediction whether the word is correct or not. For “misspelled” words, a list of candidates is also generated.

Language modeling is the task of assigning probability to sentences in a given language. In addition to assigning a probability to each sequence of words, the language models (LMs) also assign a probability for the likelihood of a given word (or a sequence of words) to follow a sequence of words. N-gram language models are still among the most popular statistical language models today. During speech recognition decoding, candidates are evaluated using both acoustic scores and LM scores. As outlined in Figure 3, based on experiments, we propose to use a word-level N-gram language model after the spell-checking model in order to generate a candidate list using beam search with a specific width. Namely, an external LM model can re-score the final list of possible candidates. All LMs in SPREAD are trained on text datasets independently from the acoustic models. We use the scalable modified Kneser-Ney language model (KenLM) [112] estimation approach for training 6 g models for all languages. KenLM is a library that implements two data structures for efficient language model queries, reducing both time and memory costs.

#### 3.2.2. From Diary Recording to Updated Insights on Patient Condition

**Figure 4:**
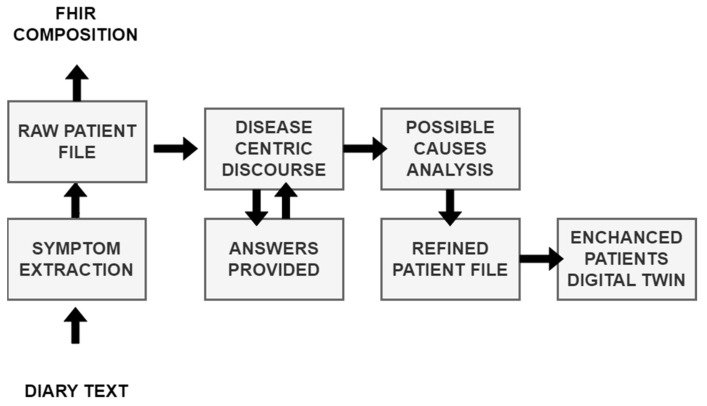
From diary recording to updated patient profile.

The main objective of MRAST is to generate additional insights on symptoms and well-being in real-life settings. Namely, multiple studies have shown that symptoms extracted from conversation can greatly improve the accuracy of disease identification and disease progression [116]. The implementation and workflow are outlined in Figure 4. 

The diary text, automatically extracted from diary recordings (see Section 3.2.1, Speech Recognition Engine) is first sent to the symptom extraction service based on Symptoma’s AI [117]. The approach aims at mining data features from free text that are medically relevant and can represent the documented content. This exploits Symptoma’s proprietary disease database built from proprietary disease concepts as well as its proprietary ontology, structuring disease—symptom—risk factor—etc. Symptoma’s proprietary ontology is developed by analyzing medical articles, case reports, and patient-generated data from 36 languages. Further, learning from billions of anonymized keywords entered by more than 10 million monthly users allows the addition of (lay) terminology variants, which is unparalleled, looking at the existing ontology landscape. The AI technology developed by Symptoma is a significant technological breakthrough, built on more than 15 years of research and development in the field. It is designed to understand the medical context of information units and to identify disease–symptom–risk factor relations, making it a valuable tool for medical professionals and patients alike. Symptoma AI boasts an accuracy rate exceeding 95% across a vast spectrum of over 20,000 diseases. The extracted concepts are stored as FHIR compositions [118] on the big data platform (See Section 3.2.3, Big Data Platform and HL7 FHIR Server). The extracted concepts are further transmitted to the “disease-centric discourse” to (1) trigger relevant PROs to be filled in or (2) to trigger a simple Q&A (with binary answers) to provide further context on the symptoms identified.

If (1) the Rasa framework-based DCD is activated to facilitate the completion of PROs, the outcomes provide valuable subjective information directly from the patients, contributing to a holistic understanding of their health status. The activation is initiated through a REST API called from the Open Health Connect (OHC) platform, prompting the patient to engage with the questionnaire. Simultaneously, the initially extracted concepts are supplied as input, enhancing the questionnaire’s relevance to the individual’s health concerns. The responses obtained from the patient are then relayed to the Symptoma endpoint through the UM REST API, facilitating a seamless integration of patient-reported data with the symptomatic information extracted from the diary recordings.

Moreover, (2) the DCD employs a tailored questionnaire designed for binary answers. This approach aims to further contextualize the concepts identified during the initial extraction process. The DCD, guided by Symptoma’s AI and considering the patient’s responses, refines its understanding of the symptoms, signs, and risk factors. This refined information is pivotal in generating an updated patient profile, enhancing the accuracy and relevance of the insights derived from the diary recordings.

As illustrated in Figure 3, the journey from diary recording to an updated patient profile embodies a dynamic feedback loop. Symptoma’s AI is not only extracting relevant data features, but also contextualizing their relations to diseases, suggesting further leading questions. This iterative process of data extraction, contextualization, and user interaction establishes a robust foundation for generating real-world insights into patient conditions and disease progression.

#### 3.2.3. Big Data Platform and HL7 FHIR Server

The big data platform is based on Dedalus’s Open Health Connect (OHC), a predecessor of Digital Connect 4 Healthcare [119]. The OHC Digital Health Platform comprises sets of components that are orchestrated together in a holistic platform. The platform enables healthcare organizations to access, cleanse, integrate, ingest, and semantically “tag” their own data held across multiple clinical and operational systems. Unlike in traditional messaging systems or stream-processing APIs, Open Health Connect enables all interfaces to be connected to and make decisions across disparate data sources in real time. Open Health Connect provides the framework and set of tools for the integration, ingestion, storage, indexing, and surfacing of patient information. OHC facilitates innovation through near real-time access to longitudinal patient information by combining data in a defined FHIR format from a wide range of systems of record. OHC can adapt to unique business results owing to our design, which is open and flexible on purpose.

The results from the MRAST framework are stored on the UM’s FHIR server, using the CASIDE data model for cancer survivorship [120]. The UM FHIR server is based on the HAPI FHIR v2 [50] using the JSON to store the resources. FHIR defines a set of resources for representing and exchanging healthcare information, and it is designed to be easy to implement and support modern web technologies. In project PERSIST, we utilize the following resources to integrate real-world data collected from the patient diaries, i.e., the diagnostic report, observation, and composition. An FHIR diagnostic report resource is used to represent and communicate the results of diagnostic investigations, such as laboratory tests, imaging studies, or other diagnostic procedures. The diagnostic report resource provides a structured representation of the key information related to a diagnostic report, including the patient, the requestor, the service provider, the date and time of the report, and the actual diagnostic results. The FHIR Observation resource is used to represent measurements or simple assertions made about a patient or other subject. It is used for capturing clinical data related to various health parameters such as vital signs, laboratory results, and other observations. Observations can cover a wide range of clinical data, including numerical measurements, categorical assessments, and coded observations. As for the composition resource in FHIR, it is used to represent a set of healthcare-related information that is a coherent set and has clinical meaning. A composition resource typically includes metadata about the composition, such as the author, date, and context, and it may contain references to other resources, such as observations, conditions, or medications, to represent a comprehensive clinical document. Compositions are often used to create structured documents, such as discharge summaries or clinical notes, that capture a snapshot of a patient’s health status at a particular point in time. In the context of the PERSIST project, we store the patient symptoms together with the patient text transcription as results from the MRAST framework to the composition. In another composition resource that is linked to the patient resource, we also store the speech, text, and facial hand-crafted features. In the third composition resource are the extracted symptoms, signs, and causes as well as patient-provided answers to the DCD questions. In the PERSIST project, the diagnostic report was linked with the diary videos that were retrieved from the patients’ smartphones. Once the process of the MRAST framework finished, we added notes in the existing resource to mark that the linked video was annotated. The observation resource contained the final depression decision result.

### 3.3. Case Study with Full Patient Journey

A patient records their daily experiences, capturing not just words but the essence of their ailments in a video diary. The UM seamlessly extracts audio from these narratives with the help of the MRAST framework. This audio journey undergoes a transformation, owing to an automated speech recognition engine. It skillfully transcribes the patient’s spoken words into text, revealing valuable information. Consider a snippet from this textual tapestry: “I have a slight fever today, my head hurts, and my throat hurts. Since I’m not feeling well, all I want to do is sleep.” Five symptoms emerge—sore throat, low fever, headache, pain, and insensitivity to pain, offering a glimpse into the patient’s world.

**Figure 5:**
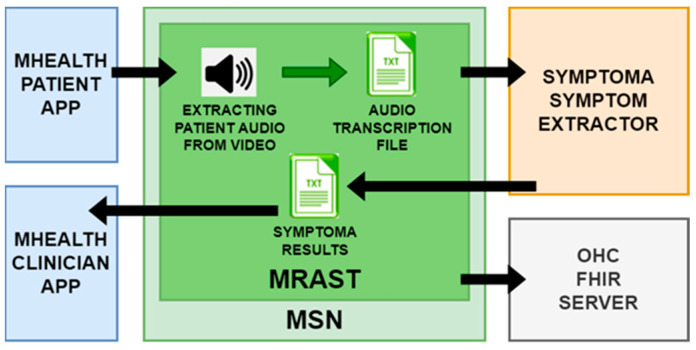
MRAST framework basic flow.

The UM has put Symptoma’s concept extraction from video diaries as the MRAST framework presented in Figure 5 to use and evaluated it. The UM pulls audio from the patient video in the flow. The automated speech recognition engine is then used to extract the transcribed text from the audio. The following sample of text has five symptoms: “I have a slight fever today, my head hurts, and my throat hurts. Since I’m not feeling well, all I want to do is sleep.” From these results, at the end as the result we can see that the sore throat, low fever, headache, pain, and insensitivity to pain are recognized as possible symptoms. Those results are then stored to a UM local FHIR server to be validated.

The identification of medical domain-specific risk factors is accomplished by leveraging Symptoma AI, which includes an AI disease engine and a sophisticated disease database. This technology is designed to analyze symptoms, signs, and risk factors gathered from millions of medical articles and publications in 36 languages, to enable the recognition of terminology variants. Symptoma AI has been extensively validated for its performance through various studies and is also utilized in the Symptoma Digital Health Assistant, a Class I medical device.

**Figure 6:**
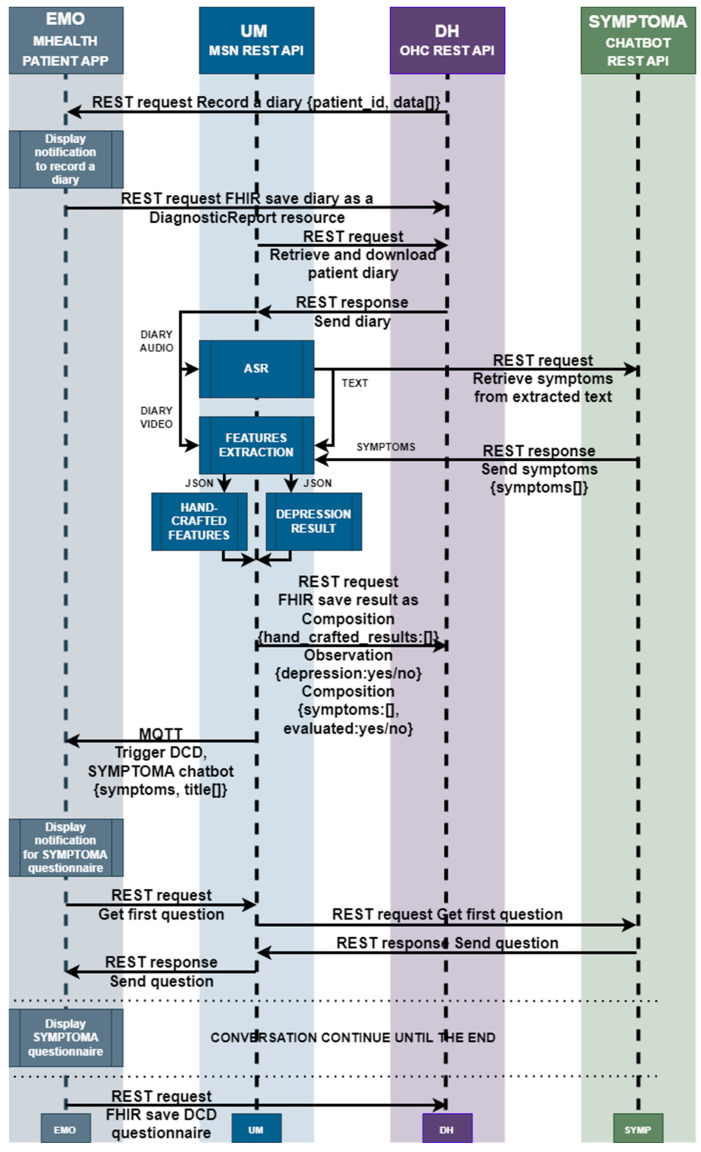
DCD communication flow of real-world implementation.

Next, we describe the real-world implementation of DCD. This section describes the step-by-step implementation of the pipeline. This pipeline implements a disease-oriented dialogue system framework to provide additional context for symptoms detected within the PERSIST MSN by conversing with patients to collect additional symptoms beyond their self-reports or the content within diaries. Figure 6 depicts an updated communication flow for the disease-centric discourse. DH is activating the flow automatically via the REST API on OHC, requesting to offer the questionnaire to the specific patient and supplying the initial patient symptom or symptoms. This is submitted to the UM REST API as a JSON payload, which is then transferred to an MQTT message with the same JSON payload.

This MQTT payload is then transmitted as a notice to the mHealth application. On the user’s smartphone, a message arrives, requesting the user to complete the required questionnaire. When the user taps on the message, the UM REST API receives a REST request. The Symptoma endpoint is then notified by UM to provide the first question for the patient. Symptoma then delivers the inquiry to the UM endpoint, which is utilized for the text-to-speech (TTS) and embodied conversational agent (ECA) machine learning (ML) microservice. This way, the UM can create the video containing the ECA video together with the TTS audio result that is presenting the retrieved question. UM transmits the received queries to the mHealth application and receives the user’s response back in the form of “YES” or “NO” answers. Symptoma receives UM’s responses, and the discussion continues until the final relevant question/answer is resolved. EMO keeps track of conversation statistics, which it will send to the DH OHC FHIR server as an FHIR composition resource. The next sections depict how this workflow works for a specific problem and for a specific questionnaire.

**Figure 7:**
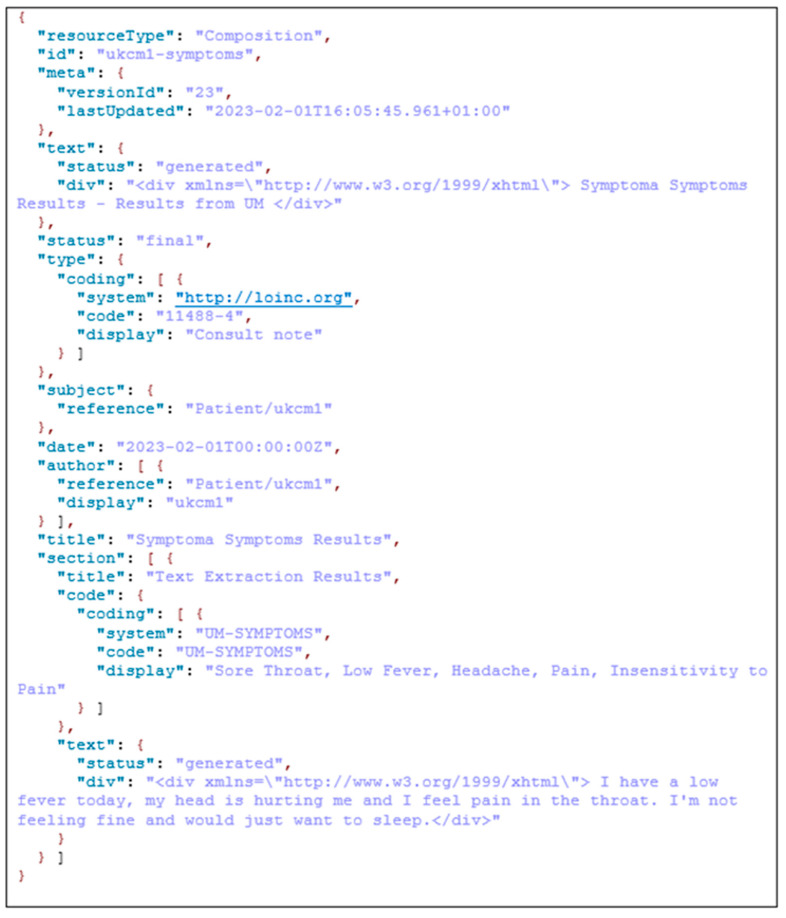
Refined FHIR composition resource including the extracted symptoms.

In Figure 7 is shown an example of the FHIR composition resource that contains the extracted symptoms and original patient text received from the speech by ASR. In the specific resource we have information about the version, so we can see how many times this resource has been updated. There is a date with a timestamp of that latest update. In the document is the reference to the patient resource to which this composition belongs. In the Symptoma’s AI results part, we have the text extraction results, and at the end is the original text input provided by the text-to-speech part over the patient voice.

## 4. Results

In this section, we provide the outcomes of our study, revealing insights across key dimensions of our framework; i.e., the multimodal sensing network (MSN) architecture, the automatic speech recognition (ASR) system, SPREAD, and the scalable MRAST framework. A real-world case study illustrates the operational context. Lastly, the PERSIST clinical trial involving 166 patients unveils valuable insights, with the system usability survey showcasing evolving perspectives on the mHealth app usability.

### 4.1. ASR Results

The ASR system SPREAD is constructed around an end-to-end deep neural model based on connectionist temporal classification (CTC), similar to models like DeepSpeech. The term “end-to-end” denotes that it relies solely on speech samples and their associated transcripts, without the need for additional information. This approach enables the system to establish a correspondence between audio and text. The ASR model within SPREAD can be distilled into two significant components: training and inference. The training of the acoustic deep learning (DL) model for SPREAD is an offline procedure.

We had enough data for training and testing in Slovenian, Latvian, English, Russian, and French language datasets. The Slovenian training took 55 days, resulting in a highly accurate and low word error rate. Similarly, Latvian training for 55 days showed exceptional performance. For English, the model trained for 81 days, demonstrating outstanding accuracy and a low word error rate. Russian training, spanning 145 days, also yielded exceptional results. Finally, French training for 185 days showcased outstanding performance with high accuracy and minimal word error rate, as shown on Table 1.

### 4.2. FHIR Server and Connectivity Tests

**Figure 8:**
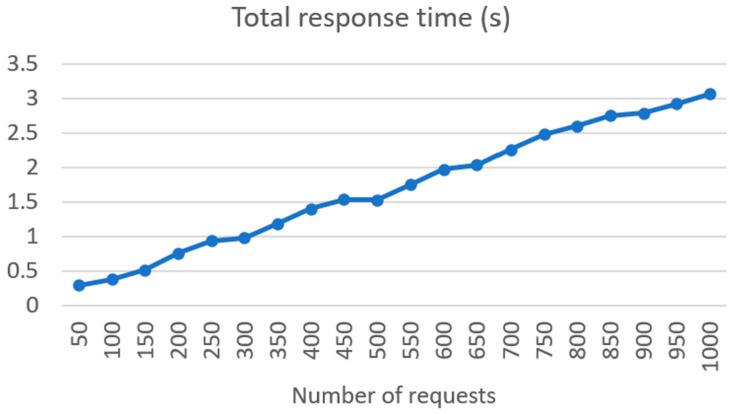
Total response time of request batches between the UM REST API and SYM symptom extractor.

The raw patient files are taken from the OHC FHIR server and processed by the MRAST framework, and the refined patient files are stored to the UM FHIR server, where they are checked for errors and then created on the OHC FHIR server. Connectivity load tests were made from UM REST API, executing the requests in batches from 50 to 1000 requests per batch. The requests included the dummy patient text with 7540 characters or 1455 words. Requests were formed and sent with the Python 3.8.12 script, wherein each request is a new thread. This way, we can see if the system would support the specific number of requests or system users, which in our case is the number of patients. Also, we can see the time needed for a specific request batch to be executed at the same time and make further decisions about future developments of the system. In the next figures, we present the results of the load tests. In Figure 8, we can see the response time in seconds based on the sent number of requests, between 50 and 1000. As we can see, the response time rises linearly from 50 requests, where we have a response time of 0.29 s, to 1000 requests, where the response time is 3.06 s.

**Figure 9:**
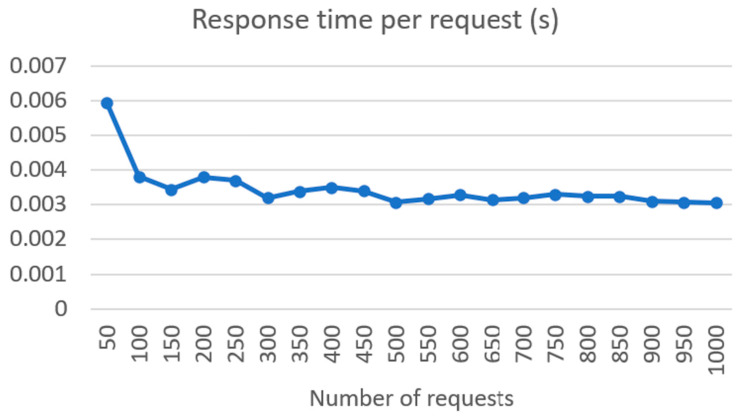
Response time for single request between UM REST API and SYM symptom extractor.

In Figure 9, we can see the response time for requests in seconds based on the sent number of requests between 50 and 1000. Here, we are observing the total response time divided by the number of requests. As we can see, the response time drops logarithmically from 50 requests, where we have a response time of 5.94 milliseconds, to 1000 requests, where the response time is 3.07 milliseconds.

**Figure 10:**
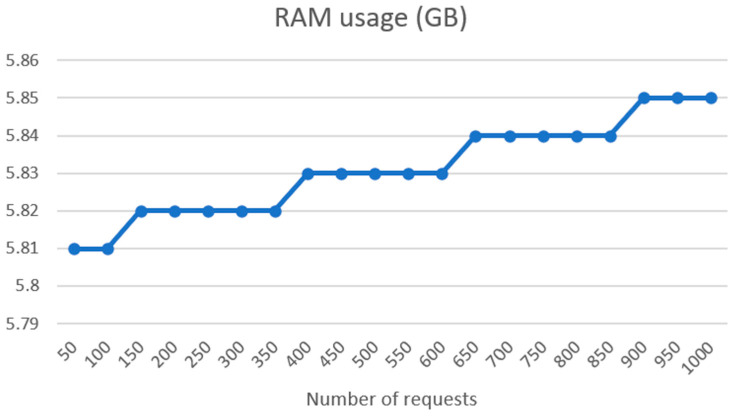
RAM usage per request batch on UM REST API side.

In Figure 10, we can see the RAM usage per request batch between 50 and 1000. RAM usage rises linearly but with very low or unnoticed rise of consumption from the 50 requests, where we have CPU requests of 5.81 GB, to 1000 requests, where RAM usage is 5.85 GB.

**Figure 11:**
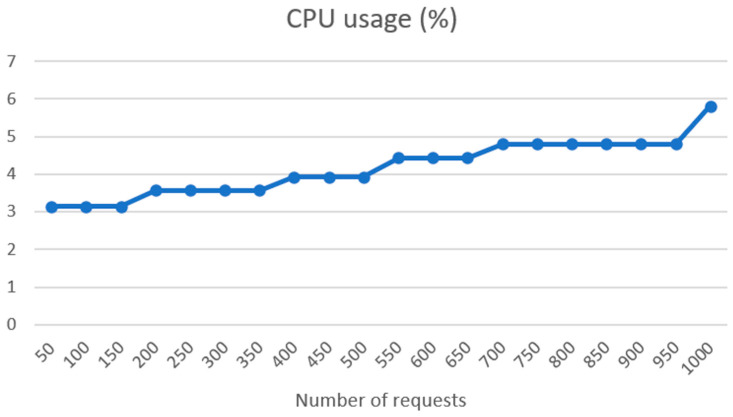
CPU usage per request batch on UM REST API side.

In Figure 11, we can see the CPU usage per request batch between 50 and 1000. CPU usage rises linearly, but with a low rise of consumption from the 50 requests, where we have CPU usage of 3.14%, to 1000 requests, where CPU usage is 5.81%.

**Figure 12:**
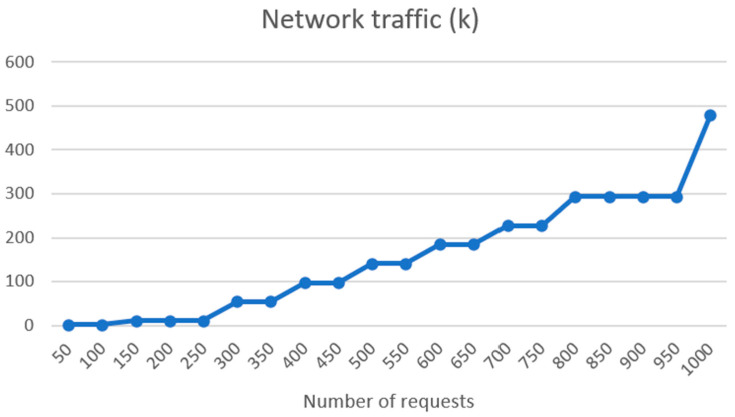
Network traffic per request batch on UM REST API side.

In Figure 12, we track network traffic per request batch between 50 and 1000. Network traffic rises exponentially from the 50 requests, where we have network traffic of 2.49 k, to 1000 requests, where network traffic is 479.5 k.

Compared with Suresh et. al. [121], the proposed system can carry out a higher number of requests in less time and have better performance, but with a smaller size FHIR resource used while making tests. In their paper, the performance testing was made where they took a specific HL7 FHIR resource questionnaire for GAD-7. They executed 50 concurrent users in over 20 min and received an average response time between 0.3 and 0.5 s.

### 4.3. Patient Evaluation

A total of 166 patients took part in the PERSIST clinical trial across four hospitals, as detailed in Table 2. Among these participants, 85 individuals were diagnosed with breast cancer, while 81 were affected by colorectal cancer. The average age of the patients upon enrollment was 55 years. The study comprised 37 male and 129 female patients, resulting in an uneven gender distribution. This disparity can be attributed to the infrequency of breast cancer in men and a slightly higher representation of women in the group of patients with colorectal cancer. This gender imbalance in inclusion is further explained by the clinicians’ observations, who noted that men displayed less interest in participating in the study compared with women.

To collect feedback from patients, app-based questionnaires named PERSIST block ABC [122] were administered at three distinct time intervals to gain insights into their participation experience in the study and to highlight their key observations. The surveys from Table 3, Table 4, and Table 5 received responses from a total of twenty participants across three time points, with four participants from CHU, four from SERGAS, and twelve from UKCM. Notably, no participants from UL responded throughout all three survey instances. Those surveys were distributed at the commencement of the app-based questionnaire (First), after the introduction of the virtual agent (Middle), and at the conclusion of the study in October 2022 (Last). Participants rated their experiences on a scale of 1 (poor) to 10 (excellent). Analysis revealed no statistically significant differences between any two time points, as indicated by the Friedman one-way repeated measure analysis of variance by ranks with a *p*-value of 0.779. Furthermore, Conover’s post-hoc pairwise comparisons demonstrated no significant differences in the *p*-values among the initial-mid (*p* = 0.490), initial-final (*p* = 0.843), and mid-final (*p* = 0.622), affirming the consistency of responses across the survey periods. Table 3 shows descriptive statistics that summarize the answers to the question, “How do you rate your experience with questionnaires in the app?”

The information presented in Table 4 summarizes answers to the question, “How do you rate your experience with the mHealth app?” That information indicates that there were no statistically significant variations in participants’ assessments of the app’s ease of use between any two time points. The outcomes from the Friedman one-way repeated measure analysis of variance by ranks indicate a non-significant *p*-value of 0.279, suggesting that observed discrepancies in the ratings were likely due to random chance. Additionally, Conover’s post-hoc pairwise comparisons underscore that there were no notable distinctions between the initial and middle time points (*p* = 0.891). However, significant differences were observed between the initial and final time points (*p* = 0.138) and the middle and final time points (*p* = 0.176).

Table 5 represents the statistics that answer the question, “How do you rate your experience with diary recording?” Table 5 reveals that Friedman one-way repeated measure analysis of variance by ranks indicated no statistically significant differences between any two time points (*p* = 0.581). Conover’s post-hoc pairwise comparisons further confirm the absence of significant differences for the initial-mid (*p* = 0.304), initial-final (*p* = 0.512), and mid-final (*p* = 0.707) time points.

SUS questionnaires [122] were provided to patients with the same approach as the PERSIST block ABC questionnaires. Participants completed the survey three times: at the start, in the middle, and at the end of the clinical study. The 27 patients finished all questionnaires all three times. For each patient, the SUS score was calculated based on their responses to the 10 questions. According to the system usability levels, at the beginning of the study, most patients perceived the system as having “usability issues” (10 responses) and being “acceptable to good” (10 responses). This perception could be linked to patients’ prior experiences with technology in general, including different types of applications, and their ability to adapt to the mHealth app, which was still under development. Throughout the study, the proportion of participants who deemed the system to have “excellent usability” rose from 14% to 33%. This improvement can be attributed to the continuous enhancements made to the mHealth app in collaboration with technical partners. By the study’s conclusion, the predominant scoring category for the system was “Experiencing usability issues.” However, despite this, 44.44% of patients rated the usability as good or excellent, combining responses of “Acceptable to good” and “Excellent usability.”

Clinicians working with the mHealth app at the four participating hospitals also received SUS questionnaires. Two rounds of responses were collected: the first round involved only the mHealth app web version, and the second round included the mHealth app mobile version at the end of the study. The findings from the SUS questionnaire distributed to clinicians utilizing both the mHealth web and mobile app versions indicate that a majority of clinicians identified some usability issues (81.55% in the first round and 87.5% in the second round). However, it is noteworthy that the scores did not significantly differ between the two rounds, suggesting that the introduction of the app version did not bring about new usability issues.

### 4.4. Feasibility of MRAST Framework in the Real World

Table 6 represents a comparison of the project PERSIST study with similar studies that provided mHealth applications and similar systems for use to patients and then evaluated the overall user experience, usability, and general feedback of using the mHealth app. In the table we add the included studies and which questionnaires they used to collect the patient feedback. We present the number of patients included in each study and patient feedback in a range from strongly negative to strongly positive. All studies showed positive to strongly positive patient feedback and grades of the apps.

Short et. al. [117] performed an evaluation with 10 cancer patients. On a scale from 1 (strongly negative) to 5 (strongly positive) patients graded the app with a high grade of 4.4, which was a little bit higher than the grade of the patients in our study, but we used a bigger patient sample size. The apps that were suggested were typically perceived as user-friendly, with a grade of 4.1; the process of aligning apps with participant preferences was viewed as beneficial and was graded as 4.2. Nonetheless, all results show that the patient feedback was generally positive. Loh et. al. [102]’s primary outcome was usability assessed by the system usability scale (SUS). Their study included 18 cancer patients and 13 caregivers on a scale from 1 (strongly negative) to 5 (strongly positive). A significant portion of patients and caregivers expressed appreciation for and enjoyed the experience, recognizing the value of the proposed app. The overall satisfaction using the app for patients was graded 3.4, while the other question in the SUS varied from grade 3.0 to 3.6. Moorthy et. al. [103] had 133 cancer patients using their app and grading it in a range from 0 to 100. In that study, descriptive statistics were analyzed for the SUS and MAUQ (mobile app usability questionnaire) to evaluate the usability of their app. The participants demonstrated a notably high perceived usability, as evidenced by the SUS score with a mean of 88.3, surpassing the average score of 68. Similarly, the MAUQ produced a mean score of 85.89, providing additional confirmation of the positive perception of usability. In the study of Teckie et. al. [104], out of the 32 participants eligible for analysis, 53% (17) completed all scheduled sessions, 63% (20) completed 75% or more, and 78% (25) completed at least 50% by the study’s end. At the study’s conclusion, 53% (17/32) reconsented for SUS. The mean SUS score (95% CI) was 71.9 (64.3–79.5), indicating an “acceptable” rating. Subscale analysis revealed the learnability domain mean (95% CI) as 78.7 (71.2–86.1) and the usability domain mean (95% CI) as 70.2 (61.8–78.7). In the SUS responses, 88% found their app “easy to use,” 94% believed most could learn it quickly, and 82% felt very confident. Regarding usefulness, 76% agreed, with 59% and 71% agreeing with PRO frequency and length. Additionally, 76% would recommend their app, and 29% provided feedback, using words like “informative,” “helpful,” and “valuable” to express their positive experience. In the study by Paulissen et. al. [105], a total of 15 cancer patients returned SUS forms that were used in the analysis of this study which graded the mHealth app in range from 0 to 100. The outcomes from the computed SUS scores indicate a mean score of 86.8, categorizing the mHealth app as excellent. Participants found it helpful to respond to health questions before their appointments using the application. They mentioned that it allowed them to discuss all health issues more thoroughly during their visit, making it a more efficient and effective experience within the allotted time. This aligns with other compared studies, where the use of mHealth apps is generally acknowledged. Questionnaires from our study show that the patient general feedback about the mHealth app, questionnaires, and video diaries is strongly positive.

## 5. Discussion

In the realm of healthcare, the unparalleled significance of real-world data, particularly in the context of chronic diseases, cannot be overstated. Traditional measures such as patient-reported experience and outcomes measures (PREMs/PROMs) often grapple with limitations stemming from subjectivity and a lack of precision in targeting the nuanced experiences of individuals facing chronic conditions. The intricate and diverse array of symptoms experienced by these patients poses a challenge in selecting appropriate questionnaires to accurately capture their multifaceted realities. This limitation is where the potential of diary recordings emerges as a promising solution. Unlike conventional measures, diaries offer a comprehensive lens into psychological well-being by encompassing both physiological and psychological symptoms. Beyond symptomatology, these records illuminate non-symptomatic aspects and lifestyle choices, providing healthcare providers with a holistic understanding of a patient’s life. Recognizing the enormous utility of such data, not only does this facilitate a more nuanced measurement of everyday experiences, but it also paves the way for patient-centered care by offering insights into aspects previously overlooked. Moreover, the automated extraction of information from diaries represents a pivotal stride toward a patient-centered healthcare paradigm where decisions are rooted in real-world experiences and tailored to individual needs. Embracing this approach holds the potential to revolutionize healthcare practices and substantially enhance patient outcomes.

The good performance of the ASR system, SPREAD, across multiple languages is noteworthy. The substantial training durations for the models indicate the depth of learning, and the low word error rates (WERs) affirm the accuracy achieved. The results highlight the efficacy of end-to-end deep neural models based on CTC, emphasizing their potential for real-world applications in diverse linguistic environments. The integration of Symptoma’s AI showcases a significant advancement in medical domain-restricted risk factor extraction and opens the possibilities of predictive precision medicine. The utilization of AI for identifying and standardizing medically relevant concepts from free text makes unstructured data comparable, thus laying the foundation for building predictive models on top. The successful implementation of the FHIR server, coupled with the results of connectivity load tests, underscores the system’s robustness. Linear increases in response time and RAM usage, along with low CPU usage increase, indicate efficient handling of requests. The exponential rise in network traffic illustrates the scalability of the system, a crucial aspect for handling a substantial number of patients in real-world scenarios.

There are some drawbacks and limitations of the MRAST framework that should be considered if taking this approach in practice. The first one is the problem with the use of the diary recording functionality in the mHealth app. During the initial tests, some patients had difficulties with the use of some of the diary functionality. In order to improve the user experience of a mobile health (mHealth) app, co-creation sessions were conducted, resulting in the integration of specific elements such as a frame showcasing the position of the face, pause functionality, and an automatic end to the recording after 5 min of inactivity. The impact of these modifications and improvements was highlighted by the continuous increase in perceived experience with the app. Future research should focus on investigating the functional perspectives of mHealth apps, with a specific emphasis on introducing tasks into the everyday life of patients as nonintrusively as possible [123]. The second limitation relates to the errors in speech recognition. Namely, although with continuous retraining of the acoustic model the WER decreases, the WER might not be a realistic representation of what happens in the wild [124]. As also observed during the initial tests in real-world environments, the challenges posed by factors such as background noise, speaker variations, and the presence of multiple dialects have significant impacts on the actual accuracy of the model, e.g., the batch WER vs. test WER in Table 1. To correct misspellings and grammatical errors, we added a language model and spell-checking model to correct ASR errors. Overall, the final model achieves real accuracy over 92%, even for non-mainstream languages, which is comparable to the existing state of the art. However, future research should continue to enhance the accuracy and precision of speech recognition systems, particularly in the presence of background noise, various accents, and dialects [125]. Furthermore, as also highlighted in this paper, advancements in natural language understanding can enable more seamless and intuitive communication, as well as the integration of multimodal sensing for improved accuracy [126].

As observed in the results, the distributed architecture deployed in the experiments may cause certain delays in the overall execution. Depending on the availability of system resources and network traffic, our simulations already show that the responsiveness of the system may quickly traverse from real time, with execution times in milliseconds, to interactive time, with execution in several seconds. However, the benefits of distributed architecture with multiple networks carrying out specific tasks include overall performance and scalability, reliability and resilience, and efficiency (e.g., resource sharing, use of energy-efficient hardware, etc.). Finally, in the current use of the pipeline, the delays did not present a specific relevance or limitation. Namely, our approach was to represent the results to clinicians as background used during follow-up. If, however, similar systems should be used in concepts such as prescreening, research should focus on developing efficient communication protocols and network optimization techniques to minimize delays and ensure reliable data transmission within distributed systems [127].

The patient evaluation in the PERSIST clinical trial provides insights into the real-world usability and acceptance of the integrated technologies. Participants generally expressed a positive outlook toward the app-based questionnaires, yielding a mean score of 7.48 across all time points (initial, middle, and final). The median score of 8 indicates that most participants rated their experience as either “good” or “excellent.” The relatively low standard deviation implies minimal variability in participants’ ratings. Moreover, the absence of statistically significant differences between any two time points suggests that participants’ perceptions of their questionnaire experience remained relatively stable over time. This consistency implies that the app maintained a steady level of usability and effectiveness throughout the study duration. In summary, the data indicates a favorable participant experience with the app-based questionnaires. However, it is crucial to recognize that this assessment represents just one facet of the app’s performance. Further research may be necessary to comprehensively assess its effectiveness and user-friendliness. Respondents to this inquiry typically reported a favorable encounter with the diary recording in the application, giving it an average rating of 7 out of 10. The median rating remained consistent at 8 out of 10 for both the middle and final time assessments, suggesting a sustained positive experience for these individuals over time. Furthermore, there were no statistically significant differences observed between any two time points, indicating the stability of the positive experience with diary recording for these participants throughout the study.

In general, patients consistently evaluated their experience with the mHealth app positively, and these ratings exhibit a slight upward trend over time. Importantly, there are no statistically significant differences observed between any two time points, signifying the app’s consistently favorable reception among patients throughout the study. Notably, the middle test saw patients from all centers awarding higher scores, indicating the app’s consistent utility across diverse locations. Despite CHU patients providing the lowest ratings, the absence of statistically significant differences implies that the app was generally well received irrespective of the specific center.

The use of AI technologies in healthcare, such as Symptoma AI and chatbots, also presents some challenges and ethical dilemmas. These include privacy concerns and data bias. Patients may have concerns about the confidentiality of their information shared with the MRAST framework. AI language models, including chatbots, are susceptible to various biases, which can compromise the accuracy and fairness of medical information.

The utilization of an MRAST framework for the collection and storage of patient data raises specific privacy and security concerns. Patients may disclose sensitive health information during interactions with the MRAST framework, leading to the inclusion of this information in the FHIR database. The security and confidentiality of patient records, known as medical privacy or health privacy, are paramount and require robust safeguards to prevent unauthorized access or disclosure. It is also crucial to use as little identifying data as possible and to seek patient consent for data usage. In cases where consent is not feasible, special permissions and ethical approvals are required to use patient data for research or analysis. Personal data breaches, including unauthorized access or disclosure, must be addressed in accordance with legal requirements.

By leveraging Symptoma AI to extract and analyze symptoms from patient video diaries, the MRAST framework can offer numerous benefits. The MRAST framework can aid in the early recognition of potential symptoms by analyzing patient-provided data, enabling better treatment. It can contribute to more accurate symptom analysis, leading to improved and more tailored treatment plans. This solution was created for and tested by 166 oncology patients without looking at their socioeconomic backgrounds. By facilitating remote symptom analysis, the MRAST framework can improve access to care for patients, particularly those from diverse linguistic and socioeconomic backgrounds who may face barriers to traditional healthcare services. The framework’s ability to identify symptoms and potential causes using AI and chatbot technology can support the development of personalized care recommendations, thereby improving patient quality of life.

## 6. Conclusions

In conclusion, the work presented in this paper represents a significant step forward in enhancing patient evaluation and real-world data collection. The ASR system demonstrates robust performance across various languages, with the use of Symptoma’s AI extracting medical risk factors from free text and contextualizing them in relation to possible causes. The successful implementation of the FHIR server ensures seamless connectivity and scalability. The patient evaluation underscores the importance of continuous improvement in mHealth apps. The outcomes of the PERSIST clinical trial, combining advanced technologies and patient-centric approaches, provide a foundation for future developments in healthcare. The discussed technologies offer promising avenues for improving diagnostics, patient engagement, and overall healthcare outcomes. As technology continues to evolve, ongoing research and refinements will further enhance the integration of ASR and AI-driven chatbots in clinical settings, contributing to more effective and patient-friendly healthcare solutions. The article identifies some issues that were resolved during study and some with which we will deal in our future studies, such as problems with recording diaries in the mHealth app, errors in ASR results, slow execution time from diary input to symptom retrieval, and engagement-related issues. Improving the video diary recording through co-creation significantly reduced technical glitches and enhanced the user experience. The accuracy of ASR was significantly improved by coupling it with natural understanding modules (i.e., spell-checker and the language model). To sum up, the outcomes underscore the MRAST framework’s potential to enhance patient quality of life and provide clinicians with deeper insights into patients’ daily experiences with their illnesses. Our future research will focus on how technology is changing healthcare and affecting how patients become involved in their care. We aim to develop creative methods that improve patient care and give useful information to doctors. Using advanced technologies, patient-focused methods, and the MRAST framework is essential for shaping the future of healthcare research and practices.

## Figures and Tables

**Table 1:** Language parameters for testing and training.

Language	Training Data	Testing Data	Training Time	Model Size	Platform	Batch WER	Test WER
Slovenian	336.74 h	85.84 h	55 days	2.6 GB	HPC GPU 2xRTX8000	0.0032%	2.3%
Latvian	782.65 h	197.08 h	93 days	2.6 GB	HPC GPU 2xRTX8000	2.03%	0.35%
English	1272.87 h	319.97 h	81 days	2.6 GB	HPC GPU 8xA100	0.7%	2.92%
Spanish	1272.87 h	319.97 h	81 days	2.6 GB	HPC GPU 8xA100	0.7%	2.92%
Russian	2796.00 h	709.42 h	145 days	2.6 GB	HPC GPU 6xA100	9.1%	2.7%
French	1272.48 h	335.49 h	185 days	2.6 GB	HPC GPU 4xV100	5.3%	7.6%

**Table 2:** General description of patients.

Clinical Partner	Recruited Patients	Mean Age	Breast Cancer	Colorectal Cancer	Male	Female
UL	46	54	24	22	7	39
UKCM	40	57	20	20	11	29
CHU	41	55	21	20	7	34
SERGAS	39	56	20	19	12	27
TOTAL	166	55	85	81	37	129

**Table 3:** Descriptive statistics of the rate of user experience (1–10) with questionnaires.

	First	Middle	Last
Mean	7.600	7.250	7.600
Median	8.000	8.000	8.000
Std. deviation	1.635	2.023	1.789
Minimum	5.000	2.000	4.000
Maximum	10.000	10.000	10.000
25th percentile	6.000	6.750	6.000
50th percentile	8.000	8.000	8.000
75th percentile	8.250	8.000	9.000

**Table 4:** Descriptive statistics of the rate of user experience (1–10) with the mHealth app.

	First	Middle	Last
Mean	7.600	7.350	7.900
Median	7.500	8.000	8.000
Std. deviation	1.667	1.899	1.553
Minimum	5.000	3.000	5.000
Maximum	10.000	10.000	10.000
25th percentile	6.000	6.000	7.000
50th percentile	7.500	8.000	8.000
75th percentile	9.000	8.250	9.000

**Table 5:** Descriptive statistics of the rate of user experience (1–10) with diary recordings.

	First	Middle	Last
Mean	6.650	7.000	7.000
Median	7.000	8.000	8.000
Std. deviation	2.455	2.753	2.695
Minimum	1.000	1.000	1.000
Maximum	10.000	10.000	10.000
25th percentile	5.750	6.750	6.000
50th percentile	7.000	8.000	8.000
75th percentile	8.000	9.000	9.000

**Table 6:** Comparison of PERSIST with other studies for cancer patients using mHealth apps.

Study	Questionnaires	Patient Sample	Patient Feedback
Short et. al. [101]	Self-defined	10	Strongly positive
Loh et. al. [102]	SUS	18	Positive
Moorthy et. al. [103]	SUS, MAUQ	133	Strongly positive
Teckie et. al. [104]	SUS	17	Positive
Paulissen et. al. [105]	SUS	15	Strongly positive
PERSIST [122]	SUS, Self-defined	166	Strongly positive

## Data Availability

The data are not publicly available due to restrictions that apply to the availability of these data.
